# Clinical Effect and Mechanism of Yisui Shengxue Granules in Thalassemia Patients with Mild, Moderate, or Severe Anemia

**DOI:** 10.1155/2016/1713897

**Published:** 2016-02-02

**Authors:** Yan-Ling Cheng, Xin-Hua Zhang, Yu-Wen Sun, Wen-Juan Wang, Su-Ping Fang, Zhi-Kui Wu

**Affiliations:** ^1^Laboratory of Molecular Biology, Guang'anmen Hospital, China Academy of Chinese Medical Sciences, Beijing 100053, China; ^2^Department of Hematology, 303rd Hospital of PLA, Nanning 530021, China

## Abstract

Yisui Shengxue granules, which is a Chinese traditional medicine, can increase hemoglobin, red blood cells, and Ret of thalassemia patients with mild, moderate, and severe anemia and thus relieve clinical anemia symptoms. Studies on mechanism found that Yisui Shengxue granules can increase the proliferation ability of hematopoietic stem cells. Emodin promoted colony forming of hematopoietic stem cells. Yisui Shengxue granules can increase the activity of GSH-PX in bone marrow blood and decreased the severity of inclusion bodies on the cytomembrane of RBCs. YSSXG attenuated anemia symptoms in patients with thalassemia mostly by increasing the proliferation of hematopoietic stem cells and decreasing the hemolysis of RBCs.

## 1. Introduction

Thalassemia is a type of hereditary hemolytic anemia, resulting from decreased expression of *α* or *β* globin genes. The most common types in China are alpha and beta thalassemia. Alpha thalassemia occurs with absence or mutation of *α*_1_ or *α*_2_ gene on chromosome 16, and beta thalassemia occurs with mutation of the *β* globin gene on chromosome 11 [1–3]. Hemoglobin (Hb) content determines the different clinical symptoms observed in thalassemia patients. In China, thalassemia is most common in the southeast area, especially in Nanning, Guangxi province, with an incidence rate of 15% [4]. The incidence rate of thalassemia in Northern Thailand and Hong Kong is 30–40% [5] and 3–5% [6], respectively.

Majority of the patients with thalassemia require lifelong blood transfusions [7] and iron chelation therapy [8, 9]. Only a few patients have the opportunity for allogeneic transplantation of hematopoietic stem/progenitor cells, which is a curative but potentially hazardous therapy [10]. Conversely, patients typically have little knowledge of gene consulting and prenatal diagnosis, which contributes to a high frequency of this genetic disease [11].

Yisui Shengxue granules (YSSXG) are a traditional Chinese medicine that has been used as an alternative therapy for almost 30 years (since 1989). It is the first and only traditional Chinese herbal medicine for the management of thalassemia. In 2006, a patent was awarded to YSSXG (number ZL200610078866X), which contains eleven components:* Cornus officinalis* Sieb. et Zucc.,* Polygonum multiflorum* Thunb.,* Rehmannia glutinosa* Libosch.,* Astragalus membranaceus* (Fisch.) Bge. var. mongholicus (Bge.) Hsiao.,* Codonopsis pilosula* (Franch.) Nannf.,* Angelica sinensis* (Oliv.) Diels,* Psoralea corylifolia* L.,* Equus asinus* L.,* Spatholobus suberectus* Dunn.,* Trionyx sinensis* Wiegmann, and* Amomum villosum* Lour. A standard quality control of YSSXG has been previously described, including identification of five main herbal components of YSSXG by thin-layer chromatography and characterization of YSSXG by high-performance liquid chromatography [12–14].

YSSXG can relieve some anemia-related clinical manifestations, including dizziness, palpitations, sallow complexion, tinnitus, and shortness of breath [15]. Previous clinical studies observed the clinical effect of YSSXG according to different genotypes. This study aimed to observe the clinical effect of YSSXG on thalassemia patients with mild, moderate, or severe anemia and to determine the possible mechanism.

## 2. Materials and Method

### 2.1. Sample Selection

Eighty-seven thalassemia patients diagnosed according to “Diagnosis and Curative Effect Evaluation Standard of Hematopathy” [16] were recruited from the Department of Hematology, 303rd Hospital of the People's Liberation Army in 2012. The Research Ethics Committee of Guang'anmen Hospital approved this clinical trial (2011NO.078). The clinical trial was performed in accordance with the principles outlined in the Declaration of Helsinki. Patients signed an informed consent and in case of minors, consent was obtained from their guardians.

The study identified 55 male and 32 female patients with an average age of 13.87 ± 8.00 (5~34). Of all the patients recruited, 21 had mild anemia, 56 had moderate anemia, and 10 had severe anemia (Table 1). The distribution of thalassemia genotypes is listed in Table 2.

### 2.2. YSSXG Doses

Patients were administered YSSXG (ratification number 20120516) for three months. Each packet of granules contained 10 g powder (1 g powder contains 2.368 g crude drug). The granules were dissolved in warm water and taken orally [17].

Emodin used in the in vitro colony forming assay experiment was a purified component from YSSXG, which was dissolved in DMSO (37 mM) and stored at −20°C. Repeated freezing and thawing before use were avoided.

### 2.3. Curative Effect Observation

An increase in Hb content of more than 5 g/L is considered to indicate effective outcome; Hb content >20 g/L is regarded as a significant curative effect. Clinical parameters: Hb; red blood cell (RBC); reticulocytes (Ret) of peripheral blood were recorded before and after one-, two-, and three-month treatment.

### 2.4. Separation of Bone Marrow Hematopoietic Stem Cells (CD34+ Cells)

Bone marrow blood was collected from the posterior superior iliac spine (ps.i), and CD34+ cells were separated using a magnetic separation method (Miltenyi Biotec CD34 Micro Bead Kit, human, number 130-046-702) from nucleated cells after density gradient centrifugation (Lymphocyte Separation Medium, TBD, number LTS1077, Tianjin, China). All stages of the experiment were conducted in a sterile environment.

### 2.5. Proliferation of CD34+ Cells

CD34+ cells were obtained from five patients before and after three months of treatment (Table 3). CD34+ cells were cultured in complete medium containing Iscove's Modified Dulbecco's Medium (IMDM; Gibco, 12440-053), 15% fetal bovine serum (FBS; TBD, D0110HYT), 100 *μ*M 2-mercaptoethanol (AMRESCO 2617B036), 50 ng/mL stem cell factor (SCF; Invitrogen, PHC2115), 50 ng/mL GM-CSF (Invitrogen, PHC2015), 5 ng/mL interleukin-3 (Invitrogen, PHC0034), 2 U/mL EPO (Invitrogen, PHC9634), 100 UI/mL penicillin (Cyclone, SV30010), and 100 mg/mL streptomycin (Cyclone, SV30010).

Cells were resuspended and adjusted to a concentration of 1 × 10^5^ cells/mL. A 96-well plate was prepared with 100 *μ*L complete medium containing cells in each well, and 6 duplicated wells were prepared for each sample. Cells were incubated at 37°C and 5% CO_2_ in a humidified incubator without disturbance. CCK-8 reagent (10 *μ*L) was added to each well received according to the instructions on the kit (Oxygenation TECH, KGA317) on days two, five, six, and seven. After 2-hour incubation, OD value was detected at a wavelength of 450 nm using an enzyme standard instrument (BioTeSYNERGY2) to observe the proliferation ability of CD34+ cells.

### 2.6. Colony Forming Assay of CD34+ Cells with Emodin

A hematopoietic colony forming assay was performed using methylcellulose semisolid medium (MethoCultTM H4034 Optimum, Stemcell). CD34+ cells were obtained from human donors, cultured in three groups with or without emodin (0 *μ*M, 4 *μ*M, and 9 *μ*M), and incubated in 24-well culture plates at 37°C and 5% CO_2_ in a humidified incubator without disturbance. The emodin concentration was referenced in the previous work [18]. Each group had three duplicate wells. Colony forming unit-erythroid (CFU-E), burst forming unit-erythroid (BFU-E), colony forming unit-granulocyte, macrophage (CFU-GM), and CFU-Mix colonies were observed. Benzidine staining was used for the identification of erythroid differentiation.

### 2.7. Detection of Hemolysis-Related Cell Cytokines

We obtained plasma before and after three-month treatment with YSSXG from both the bone marrow and peripheral blood by using a density gradient centrifugation method (Lymphocyte Separation Medium, TBD, number LTS1077). Superoxide dismutase (SOD), malondialdehyde (MDA), and glutathione peroxidase (GSH-PX) were detected with an enzyme-linked immunosorbent assay.

### 2.8. Observation of Inclusion Bodies in Erythrocytes

RBCs were separated from 100 *μ*L of heparin-anticoagulated whole blood via centrifugation at 2500 r/min for 25 minute. The packed RBCs were washed twice with two volumes of phosphate buffered saline at pH 7.3 and fixed with 2.5% glutaraldehyde at 4°C for 2 hours. RBCs were subsequently dehydrated in a series of alcohol washes (50%, 70%, 80%, and 90%, each at 4°C for 15 minute; 100% at room temperature for 10 minute), and a 100% acetone wash for 10 min twice. Finally, the cells were embedded in epoxy resin (EPON812). Samples were sliced into 50 nm ultrathin sections using LKB-V-type ultramicrotome (Sweden), mounted on uncoated copper grids, and double stained with uranyl acetate and lead citrate. The inclusion bodies of the RBCs were observed under a JEM-1010 electron microscope (Japan) at ×8000 multiples [17].

### 2.9. Statistical Analysis

Data were entered in SPSS statistical software (v.11.5). Descriptive data were expressed as mean ± standard deviation. Comparison of data before and after treatment was analyzed by a paired-samples *t*-test if the difference value(d) followed a normal distribution. If the distribution was abnormal, they were analyzed with a nonparametric test (two-related-sample tests, Wilcoxon). A Chi-Square test was used to analyze the clinical effectiveness rate. A probability value of less than 0.05 was considered statistically significant. Comparison of clinical effective rates between two groups was considered statistically significant at *p* value <0.0167.

## 3. Results

### 3.1. Outcome of Clinical Curative Effective Rate

After first month of YSSXG treatment, the clinical curative effect rates were 14.29%, 44.64%, and 70.00% in mild, moderate, and severe anemia groups, respectively. The effective rate in the three groups was statistically different (*p* = 0.007); further analysis showed that the effective rates in the moderate anemia group (44.64%, *p* = 0.014, <0.0167) and the severe anemia group (70%, *p* = 0.002, <0.0167) were higher than those in the mild anemia group (14.29%). There was no difference between moderate and severe anemia groups (Table 4).

The clinical effective rates after the second month of YSSXG treatment were 33.33%, 53.57%, and 70.00% in mild, moderate, and severe anemia groups, respectively. There was no difference in the three groups (*p* = 0.122, >0.05) (Table 5). After the third month of treatment, the clinical effective rates were 47.62%, 66.07%, and 70.00% in the mild, moderate, and severe anemia groups, respectively, with no significant difference among groups (*p* = 0.285, >0.05) (Table 6).

### 3.2. Change in Clinical Blood Parameters in the Three Groups

#### 3.2.1. Change in Hb Content

**Figure 1:**
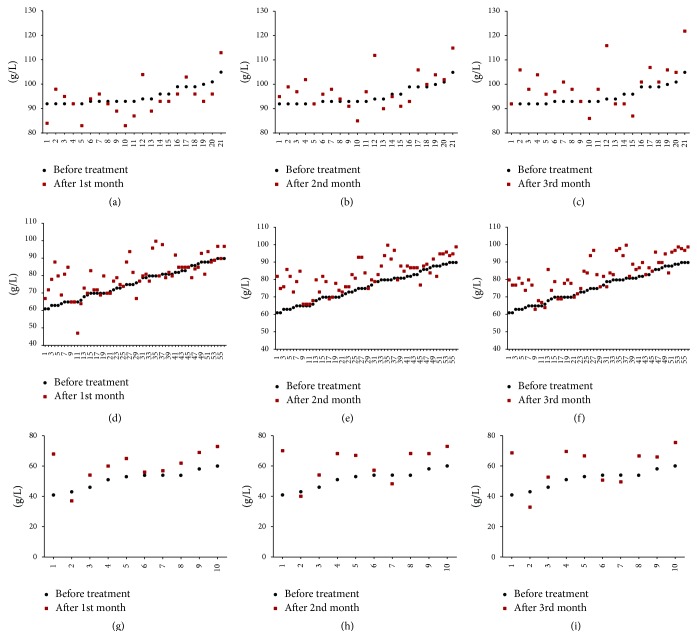
Scatterplot of the change of hemoglobin details of each patient before and after one-, two-, and three-month treatment. (a, b, and c) Change of Hb content of each patient in mild group before and after one-, two-, and three-month treatment, respectively. (d, e, and f) Change of Hb content of each patient in moderate group before and after one-, two- and three-month treatment, respectively. (g, h, and i) Change of Hb content of each patient in severe anemia group before and after one-, two- and three-month treatment, respectively.

Increase of hemoglobin after three months of treatment was presented in Table 7, and 47.62%, 66.07%, and 70.00% patients in mild, moderate, and severe group, respectively, are with Hb increase more than 5 g/L. 19.05%, 35.71%, and 50.00% patients are with Hb increase more than 10 g/L. Change of Hb content of each patient in mild, moderate, and severe anemia groups before and after one-, two-, and three-month treatment is as in Figure 1. More clinical trials on hemoglobin change treated with YSSXG beyond three months are needed.

**Figure 2:**
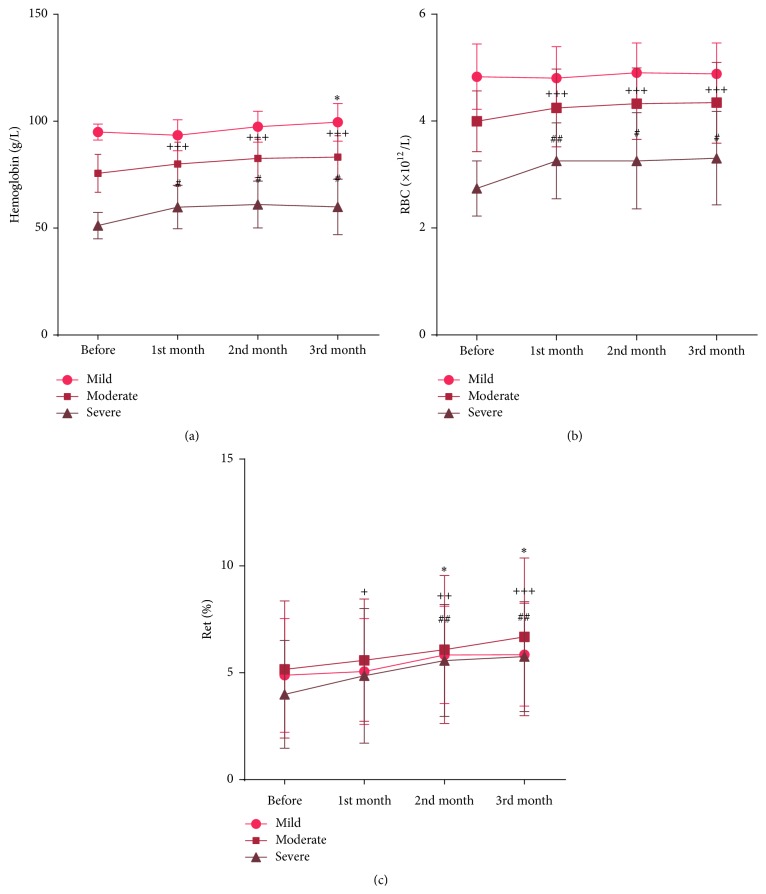
Change of clinical blood parameters of thalassemia patients in three groups (mild, moderate, and severe). (a) Change of Hb before and after one-, two-, and three-month treatment. (b) Change of RBC before and after one-, two-, and three-month treatment. (c) Change of Ret before and after one-, two-, and three-month treatment. ^*∗*^In mild group, compared with before treatment, ^*∗*^ *p* < 0.05. ^+^In moderate group, compared with before treatment, ^+^ *p* < 0.05; ^++^ *p* < 0.01; ^+++^ *p* < 0.001. ^#^In severe group, compared with before treatment, ^#^ *p* < 0.05; ^##^ *p* < 0.01.

In the mild anemia group, Hb content of blood was 95.29 ± 3.74 g/L, 93.76 ± 7.26 g/L, 97.81 ± 7.26 g/L, and 99.90 ± 8.80 g/L before and after one, two, and three months of treatment, respectively. The Hb content significantly increased after three months of treatment (*p* = 0.010, <0.05) compared to that before treatment. In the moderate anemia group, content of Hb was 80.27 ± 10.29 g/L, 82.93 ± 8.95 g/L, and 83.55 ± 9.97 g/L after one, two, and three months of treatment, respectively; Hb content significantly increased compared with that before treatment, 75.93 ± 8.86 g/L (*p* = 0.000 after one, two, and three months). In the severe anemia group, Hb content was 51.40 ± 6.22 g/L, 60.10 ± 10.18 g/L, 61.30 ± 10.97 g/L, and 60.20 ± 13.04 g/L before treatment and after one, two and three months of treatment, respectively. There is a significant difference in Hb content compared to baseline after each month of treatment: first month (*p* = 0.011, <0.05), second month (*p* = 0.013, <0.05), and third month (*p* = 0.041, <0.05) (Figure 2(a)).

#### 3.2.2. Change in RBC Count

In mild anemia group, the initial level of RBCs was 4.85 ± 0.61 (×10^12^/L), and there was no statistical change after treatment, with RBC levels of 4.82 ± 0.59 (×10^12^/L), 4.82 ± 0.59 (×10^12^/L), and 4.90 ± 0.58 (×10^12^/L), after one, two, and three months of treatment, respectively. The RBC count in the moderate anemia group was 4.26 ± 0.73 (×10^12^/L), 4.34 ± 0.67 (×10^12^/L), and 4.36 ± 0.76 (×10^12^/L), after one, two, and three months of treatment. The RBC count increased significantly compared to initial value 4.01 ± 0.57 (×10^12^/L), (*p* = 0.000 after each month). In the severe anemia group, the RBC count was 2.75 ± 0.52 (×10^12^/L) initially, 3.27 ± 0.71 (×10^12^/L) after the first month, 3.27 ± 0.90 (×10^12^/L) after the second month, and 3.32 ± 0.88 (×10^12^/L) after the third month. There was a significant difference in RBC count after one (*p* = 0.005, <0.01), two (*p* = 0.030, <0.05), and three months of treatment (*p* = 0.017, <0.05) compared to that before treatment (Figure 2(b)).

#### 3.2.3. Change in Ret Count

In the mild anemia group, baseline Ret count was 4.90 ± 2.67, 5.08 ± 2.49, 5.86 ± 2.28, and 5.87 ± 2.41 after one, two, and three months after treatment, respectively. There was a significant change in Ret count after the second (*p* = 0.030, <0.05) and third month (*p* = 0.010) compared to baseline. In the moderate anemia group, Ret count was 5.61 ± 2.87 (*p* = 0.027, <0.05), 6.11 ± 3.47 (*p* = 0.005, <0.001), and 6.71 ± 3.70 (*p* = 0.000, <0.001) after one, two, and three months of treatment, respectively, with significant increase relative to initial value (4.01 ± 0.57). In the severe anemia group, Ret count was 4.01 ± 2.53 initially, 4.88 ± 3.16 after the first month, 5.60 ± 2.63 after the second month, and 5.78 ± 2.57 after the third month. There was a significant change in Ret count after two (*p* = 0.004, <0.01) and three months of treatment (*p* = 0.003, <0.01) (Figure 2(c)).

### 3.3. Proliferation of CD34+ Cells

**Figure 3:**
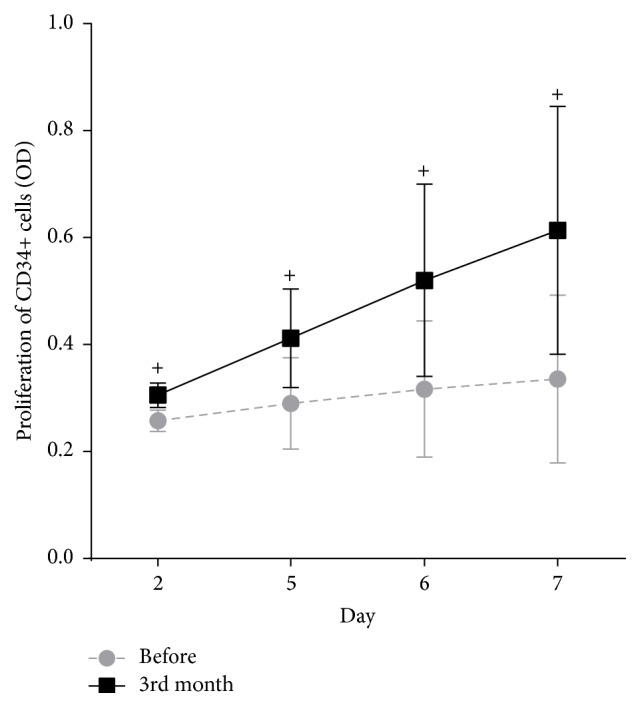
Proliferation ability of hematopoietic stem cell (CD34+) before and after three-month intervention with YSSXG.

A paired-sample *t*-test was used to analyze the changes in the proliferation ability of CD34+ cells before and after YSSXG treatment. At seven days, OD value of CD34+ cells after three months of treatment was 0.62 ± 0.23 compared to that before treatment (0.34 ± 0.16; *p* < 0.05) (Figure 3).

### 3.4. Colony Forming Ability of Hematopoietic Stem Cells with Emodin

**Figure 4:**
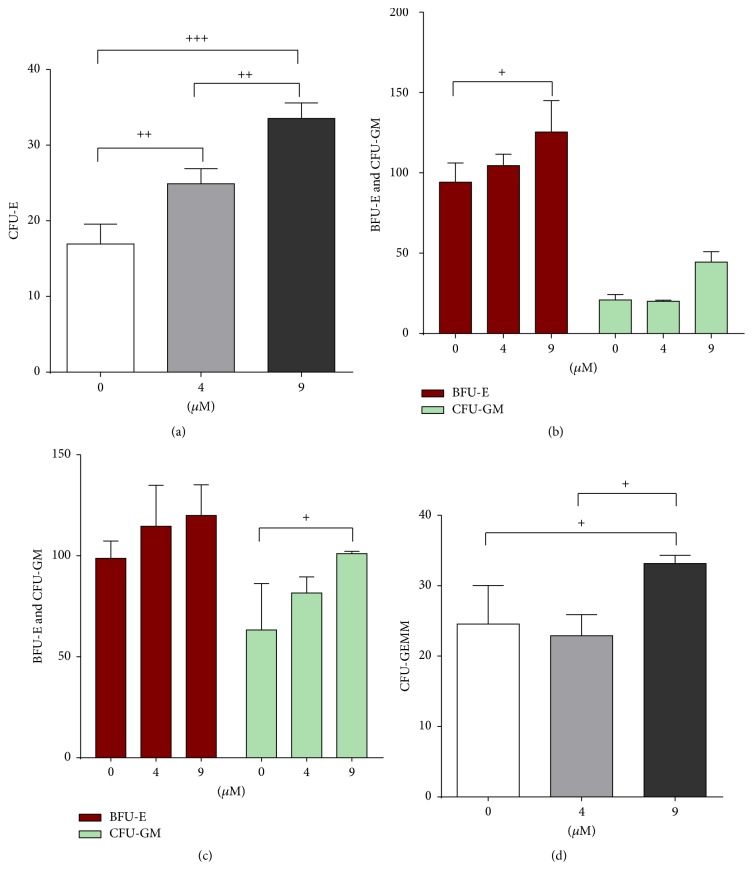
Colonies formation of hematopoietic stem cells treated with emodin. CFU-E (colony forming unit-erythroid), BFU-E (burst forming unit-erythroid), CFU-GM (colony forming unit-granulocyte, macrophage), CFU-GEMM (colony forming unit-granulocyte, erythrocyte, macrophage, megakaryocyte). (a) CFU-E after 3-day incubation. (b) BFU-E and CFU-GM after 6-day incubation. (c) BFU-E and CFU-GM after 10-day incubation. (d) CFU-Mix after 13-day incubation.

**Figure 5:**
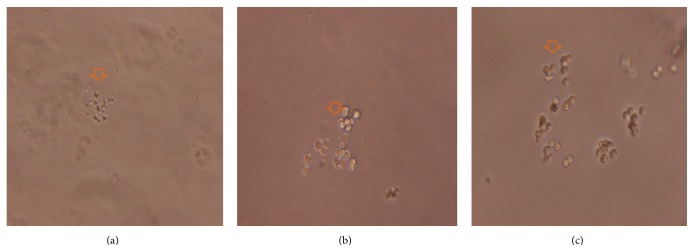
Colony forming three days after treatment. Colonies were observed under an inverted phase contrast microscope; CFU-E was indicated with orange arrow. (a) CFU-E in control group (100x). (b) CFU-E in group with 4 *μ*M emodin (100x). (c) CFU-E in group with 9 *μ*M emodin group (100x).

CFU-E colonies began forming after three days of YSSXG treatment. Colony counting showed that the number of CFU-E colonies was 33.67 ± 2.08 in 9 *μ*M emodin medium, 25.00 ± 2.00 in 4 *μ*M emodin medium, and 17.00 ± 2.65 in the control group. The number of colonies increased significantly in the 9 *μ*M emodin group (*p* = 0.000) and the 4 *μ*M emodin group (*p* = 0.005) compared with the control group. There was a significant difference in the number of colonies between 9 *μ*M emodin (*p* = 0.003) and 4 *μ*M emodin medium (Figures 4(a) and 5).

**Figure 6:**
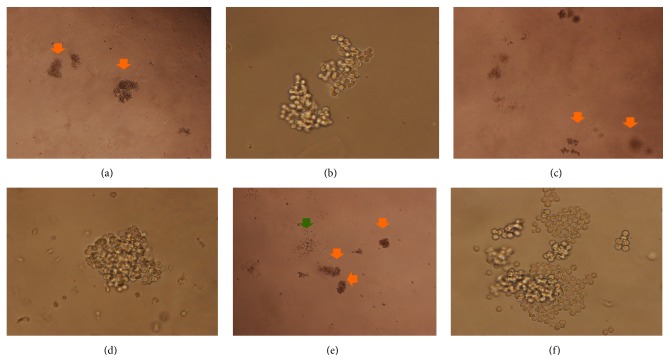
Colony forming 6 days after treatment. Colonies were observed under an inverted phase contrast microscope; BFU-E was indicated with orange arrow and CFU-GM was indicated with green arrow. (a) BFU-E in control group (40x). (b) BFU-E in control group (100x). (c) BFU-E in group with 4 *μ*M emodin (40x). (d) BFU-E in group with 4 *μ*M emodin (100x). (e) BFU-E and CFU-GM in group with 9 *μ*M emodin (40x). (f) BFU-E in group with 9 *μ*M emodin group (100x).

Colonies of BFU-E and CFU-GM began forming after six days of YSSXG treatment. The number of BFU-E colonies was 126.00 ± 19.70 in 9 *μ*M emodin medium, 105.00 ± 70.21 in 4 *μ*M emodin medium, and 94.67 ± 12.06 in control medium. The number of colonies in 9 *μ*M emodin medium increased significantly relative to control (*p* = 0.033). Hemoglobinized cells stained blue with benzidine (Figure 9). A small amount of CFU-GM began forming, and there was a difference among three groups (21.00 ± 3.46 in control group, 20.33 ± 0.58 in 4 *μ*M emodin medium, and 44.67 ± 6.51 in 9 *μ*M emodin medium) (Figures 4(b) and 6).

**Figure 7:**
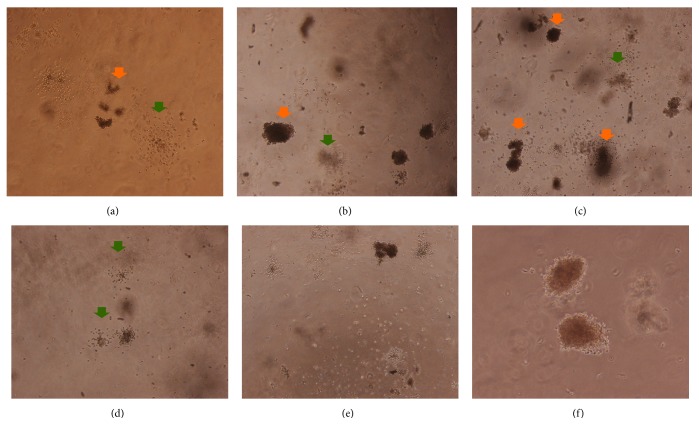
Colony forming 10 days after treatment. Colonies were observed under an inverted phase contrast microscope; BFU-E was indicated with orange arrow and CFU-GM was indicated with green arrow. (a) BFU-E and CFU-GM in control group (40x). (b) BFU-E and CFU-GM in group with 4 *μ*M emodin (40x). (c) BFU-E and CFU-GM in group with 9 *μ*M emodin (40x). (d) CFU-GM in group with 4 *μ*M emodin (100x). (e) CFU-GM in group with 9 *μ*M emodin (100x). (f) BFU-E in group with 9 *μ*M emodin, and it can be distinguished by the reddish or brownish color (200x).

After 10 days of incubation, BFU-E colonies began to mature. There was no difference among the groups (99.33 ± 8.62 in control group, 115.33 ± 20.40 in 4 *μ*M emodin medium, and 120.67 ± 15.31 in 9 *μ*M emodin medium), but CFU-GM began to increase rapidly and significantly in 9 *μ*M emodin medium (101.67 ± 1.15, *p* = 0.046), relative to the control group (67.00 ± 23.12) (Figures 4(c) and 7).

**Figure 8:**
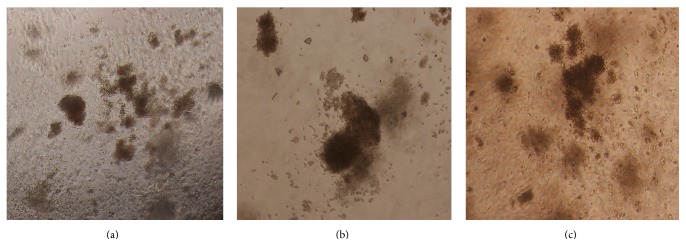
Colony forming 13 days after treatment. (a) CFU-GEMM in control group (60x). (b) CFU-GEMM in group with 4 *μ*M emodin (60x). (c) CFU-GEMM in group with 9 *μ*M emodin (60x).

**Figure 9:**
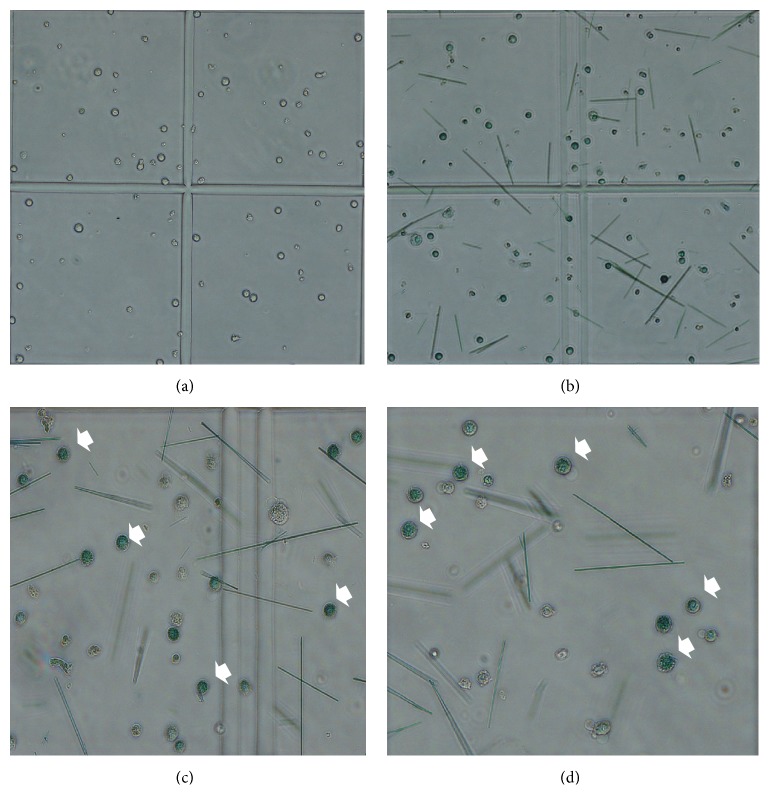
Benzidine staining to identification of erythroid differentiation. Benzidine staining positive cells were where the white arrows indicate, cells present with blue. Colonies were collected from a well using a blunt-ended needle, resuspended with PBS, and then stained with benzidine. Cells were observed under an inverted phase contrast microscope after 10 min. (a) Cells before benzidine staining. (b) Benzidine staining positive cells after staining (40x). (c) Benzidine staining positive cells after staining (100x). (d) Benzidine staining positive cells after staining (100x).

Colony forming unit-granulocyte, erythrocyte, macrophage, megakaryocyte (CFU-GEMM) colonies began forming after thirteen days, and the number of colonies in 9 *μ*M emodin group (33.33 ± 1.15) was significantly higher (*p* = 0.046) than in the control group (24.67 ± 5.51) and the 4 *μ*M emodin group (23.00 ± 3.00) (Figures 4(d) and 8).

### 3.5. Detection of SOD, MDA, and GSH-PX

**Figure 10:**
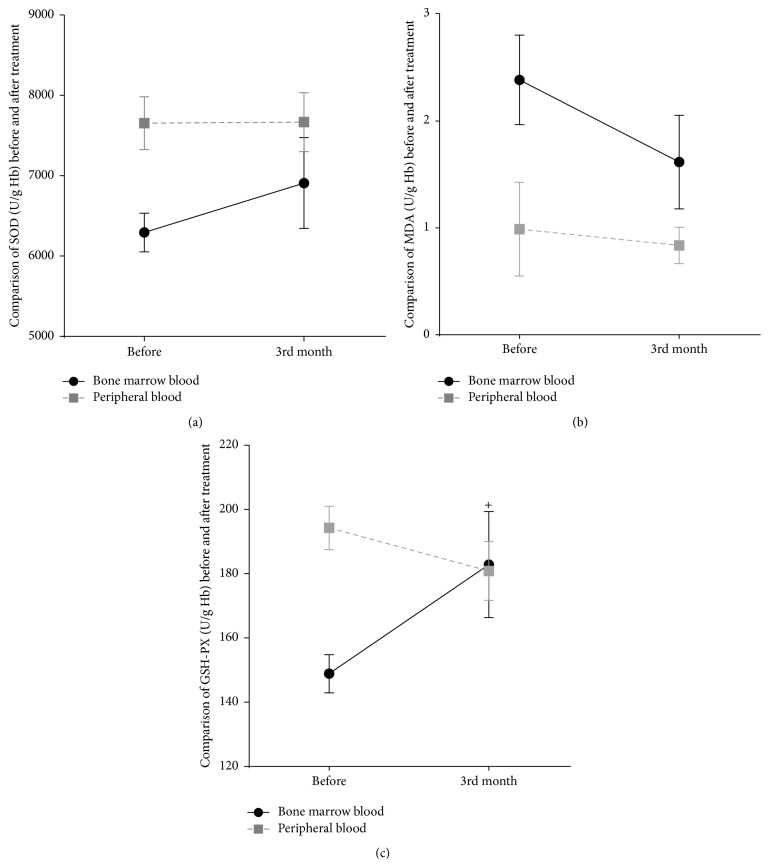
Comparison of changes in hemolysis-related cytokines before and after three months of treatment. (a) Comparison of changes in SOD activity. (b) Comparison of changes in MDA activity. (c) Comparison of changes in GSH-PX activity. Both bone marrow and peripheral blood of five patients were collected before and after treatment. A paired-sample *t*-test was used to analyze data before and after treatment, ^+^ *p* < 0.05.

There were no changes before and after treatment in SOD and MDA activity. Activity of GSH-PX in bone marrow blood significantly increased after treatment (183.11 ± 18.55) relative to baseline (149.02 ± 6.67; *p* < 0.05) (Figure 10).

### 3.6. Inclusion Bodies in Erythroid Cells by TEM

**Figure 11:**
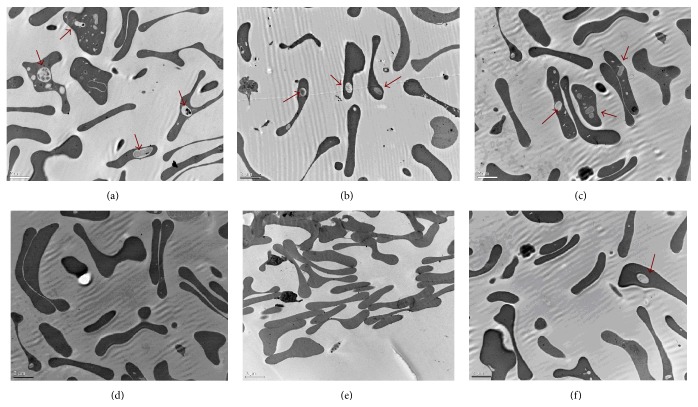
Inclusion bodies deposition in erythrocyte membrane by transmission electron microscope (TEM) (in a 0.20 *μ*m field of vision). Inclusion bodies were indicated with red arrow. (a)/(b)/(c) are inclusion bodied within red blood cells before treatment (magnification times, ×8000); (d)/(e)/(f) are inclusion bodies within red blood cells after three months of treatment.

Dark grain from results of TEM images indicated that numerous inclusion bodies formed on RBC membrane because of unmatched denatured globin chains. After three months of YSSXG treatment, the dark grains numbers decreased (Figure 11).

## 4. Discussion

YSSXG is the only traditional Chinese medicine used in the treatment of thalassemia patients. It is an alternative to the lifelong blood transfusions and iron chelation therapies, which are the standard treatments. More important, using an alternative medicine can reduce the complications of blood transfusion in thalassemia patients, such as heart and liver iron accumulation. Therefore, it is important and necessary to evaluate the effect of YSSXG in the clinical setting and to explore the possible mechanisms of this effect.

Fang's study of 156 patients with *β* thalassemia found that YSSXG could increase Hb content after treatment both in severe anemia and in moderate anemia [19]. These results showed that YSSXG had a similar clinical effect in either *α* or *β* thalassemia patients. And this Chinese medicine can relieve clinical anemia symptoms and prolong the interval between blood transfusion [20, 21].

Previous studies [19] of YSSXG to treat thalassemia focus on the thalassemia genotypes but often do not emphasize the effect in different stages of anemia. Results showed that YSSXG might be more effective in cases of moderate and severe anemia, although this finding is not statistically significant. This demonstrates the importance of considering the degree of anemia in addition to genotype when evaluating the curative effect of traditional Chinese medicine for thalassemia.

Ret is an important parameter to evaluate the proliferation ability of hematopoietic stem cells in bone marrow. Ret was 4.90 ± 2.67 in mild anemia group, 5.61 ± 2.87 in the moderate anemia group, and 4.01 ± 2.53 in the severe anemia group. The average Ret count in the three groups is 0.5–1.5% higher than the normal level, indicative of an increase in Ret count in thalassemia patients in compensation for hemolysis occurring within the body. Results showed that YSSXG can increase the percent of Ret and promote hemopoiesis.

Proliferation of CD34+ cells cultured in vitro after three months of treatment had the same results seen in the mice study. A pharmacodynamics experiment showed that YSSXG can promote hematopoietic stem/progenitor cell proliferation in mice [22]. Ma et al. [18] found that emodin could induce erythroid differentiation in K562 cells and improve the expression of globin genes. In this study, we found that emodin, an active component of YSSXG, can promote the formation of CFU-E and BFU-E colonies of CD34+ cells. These results are consistent with each other and indicate that emodin may be a key component attributable to the antianemia effect of YSSXG. Zhang and Wu [23] found that this complex prescription increased *γ*-globin, EpoR, Spi, and FKLF expression and the herbal medicine Radix polygoni multiflori can increase the Ckit expression.

For thalassemia, abnormal *α* or *β* globin genes lead to imbalance between *α* and *β* globin chains. And accumulation of relative surplus globin on the erythrocyte membrane makes the hemolysis more severe. Wang et al. [17] found that YSSXG increased the T-SOD activity and decreased inclusion bodies of RBCs after three months. We also found that YSSXG could increase the GSH-PX and decrease the inclusion bodies of RBCs.

Mechanism on YSSXG has been studied from different aspects in cellular level, and hematopoietic stem cells, K565 cells, and RBCs have been studied. Based on clinical trial and mechanism studies, we made a summary of the mechanism of YSSXG in reducing anemia in thalassemia patients, which focuses on the fact that YSSXG may promote hematopoietic ability and reduce hemolysis of red blood cells.

We had some limitations in this study. Firstly, no previous studies indicated that colony forming stimulation with YSSXG or emodin could be applied in the clinical setting; future research will use transgenic mice to evaluate the function on hematopoiesis and explore the correlation between engraftment and resultant CFU assays. Secondly, the treatment duration is three months, which is quite short considering that Chinese medicine may need a longer time to take effect; therefore, further studies should focus on the clinical effect of long-term YSSXG therapy on thalassemia and characterize possible adverse effects.

## Supplementary Material

Eleven Chinese traditional medicine constituent Yisui Shengxue granules. A standard quality control of YSSXG has been described,including identification of five main herbal components of YSSXG by thin-layer chromatography and characterization of YSSXG by high-performance liquid chromatography.

## Figures and Tables

**Table 1:** Basic information of patients with different degrees of anemia.

Groups	Cases	Age (min~max)	Gender (male/female)	Hb (g/L) (min~max)	RBC (×10^12^/L) (min~max)	Ret (%) (min~max)
Mild anemia	21	16.71 ± 8.77 (6~34)	16/5	95.29 ± 3.74 (92~105)	4.85 ± 0.61 (3.74~5.88)	4.90 ± 2.67 (1.00~12.40)

Moderate anemia	56	13.89 ± 7.85 (5~32)	33/23	75.93 ± 8.86 (61~90)	4.01 ± 0.57 (2.35~5.10)	5.18 ± 3.22 (0.60~14.00)

Severe anemia	10	7.80 ± 2.20 (5~10)	6/4	51.40 ± 6.22 (41~60)	2.75 ± 0.52 (1.92~3.48)	4.01 ± 2.53 (1.20~8.50)

**Table 2:** Genotypes distribution of patients.

Groups	Cases	Genotype of *α*	Genotype of *β*	Combined
Mild	21	*- -* ^*SEA*^ */αα* ^*CS*^ *(13); - -* ^*SEA*^ */αα* ^*QS*^ *(1);* *- -* ^*SEA*^ */αα* ^*3.7*^ *(3); - -* ^*SEA*^ */αα* ^*4.2*^ *(3)*	*β* ^17^/*β* ^*E*^	

Moderate	56	*- -* ^*SEA*^ */αα* ^*CS*^ *(34); - -* ^*SEA*^ */αα* ^*QS*^ *(1);* *- -* ^*SEA*^ */αα* ^*3.7*^ *(6); - -* ^*SEA*^ */αα* ^*4.2*^ *(4)*	*β* ^*17*^ */β* ^−*28*^ *(2); β* ^*654*^ */β* ^*E*^ *; β* ^*41-42*^ */β* ^*IVSI*-*I*^ *; β* ^*41-42*^ */β* ^−*28*^ *;* *β* ^*41-42*^ */β* ^*E*^ *; β* ^−*28*^ */β* ^−*28*^ *;* *β* ^*43*^ */β* ^*E*^ *; β* ^−*29*^ */β* ^*17*^;	*β* ^*41-42*^ */β* ^*17*^ */α* ^*WS*^ *α/αα;* *β* ^*41-42*^ */- -* ^*SEA*^ */αα* ^*3.7*^

Severe	10	*- -* ^*SEA*^ */αα* ^*CS*^ *(2)*	*β* ^*41-42*^ */β* ^−*28*^ *(2); β* ^*17*^ */β* ^*E*^ *(3);* *β* ^*41-42*^ */β* ^*E*^ *; β* ^*71-72*^ */β* ^*E*^ *; β* ^−*28*^ */β* ^−*28*^	

**Table 3:** Basic information about five thalassemia patients.

Number	Male/female	Age	Height (cm)	Weight (kg)	Genotype	Hb (g/L)
1	Male	13	161	42	- -^*SEA*^ */α* ^*CS*^ *α*	89.00
2	Male	9	125	21	*β* ^*41-42*^ */β* ^−*28*^	91.00
3	Male	9	125	21	*β* ^*41-42*^ */β* ^−*28*^	67.00
4	Male	12	133	27	- -^*SEA*^ */α* ^*CS*^ *α*	79.00
5	Male	12	130	25	- -^*SEA*^ */*-*α* ^*4.2*^	95.00

Notice: cases number 2 and number 3 are twin brothers; cases number 4 and number 5 are twin brothers.

**Table 4:** Effective rate after 1st-month treatment.

Groups	Effective	Noneffective	Total case	Effective rate (%)
Mild anemia	3	18	21	14.29
Moderate anemia	25	31	56	44.64^*∗*^
Severe anemia	7	3	10	70^*∗*^
Total case	35	52	87	40.23

Chi-Square test.^*∗*^, comparation with mild anemia group. ^*∗*^ *p* < 0.05.

**Table 5:** Effective rate after 2nd-month treatment.

Groups	Effective	Noneffective	Total case	Effective rate (%)
Mild anemia	7	14	21	33.33
Moderate anemia	30	26	56	53.57
Severe anemia	7	3	10	70
Total case	44	43	87	50.57

**Table 6:** Effective rate after 3rd-month treatment.

Groups	Effective	Noneffective	Total case	Effective rate (%)
Mild anemia	10	11	21	47.62
Moderate anemia	37	19	56	66.07
Severe anemia	7	3	10	70
Total case	54	33	87	62.07

**Table 7:** Increase of hemoglobin after three-month treatment.

Groups	*N*	Hemoglobin (g/L)				
		[~5)	[5~10)	[10~15)	[15~20)	[20~)
Mild	21	11	6	2	1	1
Moderate	56	19	17	9	10	1
Severe	10	3	2	2	2	1

## References

[1] Cohen A. R., Galanello R., Pennell D. J., Cunningham M. J., Vichinsky E. (2004). *Thalassemia*.

[2] Muncie H. L., Campbell J. S. (2009). Alpha and beta thalassemia. *American Family Physician*.

[3] Kotila T. R. (2012). Thalassaemia is a tropical disease. *Annals of Ibadan Postgraduate Medicine*.

[4] Pan H. F., Long G. F., Li Q. (2007). Current status of thalassemia in minority populations in Guangxi, China. *Clinical Genetics*.

[5] Fucharoen S., Winichagoon P. (1992). Thalassemia in Southeast Asia: problems and strategy for prevention and control. *Southeast Asian Journal of Tropical Medicine and Public Health*.

[6] Lau Y.-L., Chan L.-C., Chan Y.-Y. A. (1997). Prevalence and genotypes of *α*- and *β*-thalassemia carriers in Hong Kong—implications for population screening. *The New England Journal of Medicine*.

[7] Cao A., Galanello R., Rosatelli M. C. (1996). Clinical experience of management of thalassemia: the Sardinian experience. *Seminars in Hematology*.

[8] Chaston T. B., Richardson D. R. (2003). Iron chelators for the treatment of iron overload disease: relationship between structure, redox activity, and toxicity. *American Journal of Hematology*.

[9] Cohen A. R. (2006). New advances in iron chelation therapy. *American Society of Hematology*.

[10] Angelucci E., Pilo F., Targhetta C. (2009). Hematopietic stem cell transplantation in thalassemia and related disorders. *Mediterranean Journal of Hematology and Infectious Diseases*.

[11] Gambari R., Finotti A., Breda L. (2015). Recent trends in the gene therapy of beta-thalassemia. *Journal of Blood Medicine*.

[12] Shen M., Liu Q.-H., Kang Y.-L. (2011). Study on quality control standard of Yisui Shengxue granules. *Chinese Journal of Experimental Traditional Medical Formulae*.

[13] Sun Y. W., Zou Y., Liu L. (2013). Ultra performance liquid chromatography research for the YSSX granule. *Liaoning Journal of Chinese Traditional Medicine*.

[14] Sun Y. W., Liu Q. H., Wen J. (2014). Fingerprint chromatograms and characteristics peaks of Yisuishengxue granule. *Chinese Traditional Patent Medicine*.

[15] Wang W.-J., Wu Z.-K., Zhang X.-H. (2008). Clinical observation of Yisui Shengxue Granule in treating 25 patients with hemoglobin H disease. *Journal of Chinese Integrative Medicine*.

[16] Zhang Z., Shen T. (2007). *Diagnosis and Curative Effect Evaluation Standard of Hematopathy*.

[17] Wang W.-J., Wu Z.-K., Zhang X.-H. (2012). Effect of Yisui Shengxue Granule on the oxidative damage of erythrocytes from patients with hemoglobin H disease. *Chinese Journal of Integrative Medicine*.

[18] Ma Y.-N., Chen M.-T., Wu Z.-K. (2013). Emodin can induce K562 cells to erythroid differentiation and improve the expression of globin genes. *Molecular and Cellular Biochemistry*.

[19] Fang S., Wu Z., Zhang X. (2007). Clinical observation on YiSuiShengXue granul on treating 156 patients with *β*-thalassemia major and the molecular mechanism study. *Biological and Pharmaceutical Bulletin*.

[20] Wangli, Zhang X., Wu Z. (2008). A 3 case report of Yisuishengxue granule to prolong the interval of blood transfusion of thalassemias. *Military Medical Journal of South China*.

[21] Wang W.-J., Wu Z.-K., Zhang X.-H. (2009). Observations on after-effect duration of kidney-nourishing and marrow-replenishing therapy on 58 cases of mediterranean anemia. *Journal of Traditional Chinese Medicine*.

[22] Zou Y., Sun Y. W., Fang S. P. (2013). Effect of YisuiShengxue granules on the proliferation of hematopoietic progenitor cells in irradiated mice. *Journal of Traditional Chinese Medicine*.

[23] Zhang C., Wu Z.-K. (2008). Molecular pharmacological basis of the YiSui ShenXu Granule in *β*-thalassemia therapy. *Journal of Ethnopharmacology*.

